# Mitochondrial impairment and downregulation of Drp1 phosphorylation underlie the antiproliferative and proapoptotic effects of alantolactone on oral squamous cell carcinoma cells

**DOI:** 10.1186/s12967-023-04188-2

**Published:** 2023-05-18

**Authors:** Yafei Zhang, Bingqian Yang, Chengwei Tu, Yifan Ping, Shuhong Chen, Tong Wu, Zheyu Zhao, Yixin Mao, Zhan Yang, Zelin Cao, Jianmin Li, Kate Huang, Xi Ding, Gang Wu, Peng Zou, Zhennan Deng, Xiaoyu Sun

**Affiliations:** 1 School and Hospital of Stomatology, Institute of Stomatology, Wenzhou Medical University, Wenzhou, China; 2 School of Pharmaceutical Sciences, Wenzhou Medical University, Wenzhou, China; 3 Department of Prosthodontics, School and Hospital of Stomatology, Wenzhou Medical University, Wenzhou, China; 4 The First Affiliated Hospital of Wenzhou Medical University, Wenzhou Medical University, Wenzhou, China; 5 Laboratory for Myology, Department of Human Movement Sciences, Faculty of Behavioral and Movement Sciences, Vrije Universiteit Amsterdam (VU), Amsterdam Movement Sciences (AMS), Amsterdam, The Netherlands; 6 Department of Periodontics, School and Hospital of Stomatology, Wenzhou Medical University, Wenzhou, China; 7 School and Hospital of Stomatology, Wenzhou Medical University, Wenzhou, China; 8 Department of Oral and Maxillofacial Surgery/Pathology, Amsterdam UMC and Academic Center for Dentistry Amsterdam (ACTA), Vrije Universiteit Amsterdam (VU), Amsterdam Movement Science (AMS), Amsterdam, The Netherlands; 9 Department of Oral Cell Biology, Academic Center for Dentistry Amsterdam (ACTA), University of Amsterdam (UvA) and Vrije Universiteit Amsterdam (VU), Amsterdam, The Netherlands

**Keywords:** Oral squamous cell carcinoma (OSCC), Alantolactone (ALT), Reactive oxygen species (ROS), Mitochondrial impairment, Dynamin-related protein 1 (Drp1)

## Abstract

**Background:**

Oral squamous cell carcinoma (OSCC) is one of the most prevalent and fatal oral cancers. Mitochondria-targeting therapies represent promising strategies against various cancers, but their applications in treating OSCC are limited. Alantolactone (ALT) possesses anticancer properties and also regulates mitochondrial events. In this study, we explored the effects of ALT on OSCC and the related mechanisms.

**Methods:**

The OSCC cells were treated with varying concentrations and duration of ALT and *N*-Acetyl-l-cysteine (NAC). The cell viability and colony formation were assessed. The apoptotic rate was evaluated by flow cytometry with Annexin V-FITC/PI double staining. We used DCFH-DA and flow cytometry to detect reactive oxygen species (ROS) production and DAF-FM DA to investigate reactive nitrogen species (RNS) level. Mitochondrial function was reflected by mitochondrial reactive oxygen species (ROS), mitochondrial membrane potential (MMP), and ATP levels. KEGG enrichment analyses determined the mitochondrial-related hub genes involved in OSCC progression. Dynamin-related protein 1 (Drp1) overexpression plasmids were further transfected into the cells to analyze the role of Drp1 in OSCC progression. Immunohistochemistry staining and western blot verified the expression of the protein.

**Results:**

ALT exerted anti-proliferative and pro-apoptosis effects on OSCC cells. Mechanistically, ALT elicited cell injury by promoting ROS production, mitochondrial membrane depolarization, and ATP depletion, which were reversed by NAC. Bioinformatics analysis showed that Drp1 played a crucial role in OSCC progression. OSCC patients with low Drp1 expression had a higher survival rate. The OSCC cancer tissues presented higher phosphorylated-Drp1 and Drp1 levels than the normal tissues. The results further showed that ALT suppressed Drp1 phosphorylation in OSCC cells. Moreover, Drp1 overexpression abolished the reduced Drp1 phosphorylation by ALT and promoted the cell viability of ALT-treated cells. Drp1 overexpression also reversed the mitochondrial dysfunction induced by ALT, with decreased ROS production, and increased mitochondrial membrane potential and ATP level.

**Conclusions:**

ALT inhibited proliferation and promoted apoptosis of oral squamous cell carcinoma cells via impairment of mitochondrial homeostasis and regulation of Drp1. The results provide a solid basis for ALT as a therapeutic candidate for treating OSCC, with Drp1 being a novel therapeutic target in treating OSCC.

**Supplementary Information:**

The online version contains supplementary material available at 10.1186/s12967-023-04188-2.

## Introduction

Oral squamous cell carcinoma (OSCC) is one of the most common malignant head and neck carcinomas [1]. Each year, the number of new OSCC cases exceeds 370,000, with approximately 170,000 people dying from OSCC [2]. OSCC is initially characterized by asymptomatic hyperplastic lesions on the tongue, buccal mucosa, and gingiva [3]. The majority of cases are diagnosed at advanced stages with poor prognosis [4], high recurrence rates [5], and resistance to traditional therapy [6]. The 5-year survival rate of OSCC patients is only 60% despite the comprehensive and multidisciplinary therapeutic approaches [7, 8]. A thorough understanding of the pathological etiology of OSCC progression is urgently required for developing effective therapeutic strategies.

Oxidative stress (OS) refers to redox imbalance characterized by excessive reactive oxygen species (ROS) and reactive nitrogen species (RNS) [9]. Oxidative damage to DNA, proteins, and lipids significantly contributes to cancer formation and progression [10]. Additionally, antioxidants targeting ROS were long considered defensive weapons in preventing cancer development [11]. ROS are primarily generated in mitochondria, which is also the principal target of OS [12]. The multiple critical roles of mitochondria in cancer progression make them important targets for anticancer therapy [13].

Mitochondria-targeting therapies represent promising treatments for colon cancer [14] and breast cancer [15]. The underlying mechanisms include ROS regulation, mitochondrial DNA (mtDNA) interference, and mitophagy induction [13, 16, 17]. However, studies on mitochondrial-targeting therapy for OSCC are limited and need further exploration.

As a compound derived from *Inula Racemosa*, alantolactone (ALT) possesses numerous biological properties, including anti-inflammatory and antibacterial [18], antioxidant [19], and anticancer effects [20]. Additionally, studies have confirmed that ALT prevented cancer progression through regulation of mitochondria [21, 22]. ALT induced ROS production [23], promoted ROS-mediated mitochondrial dysfunction [21], and triggered mitochondrial-mediated apoptosis [24] in cancers involving the liver [25], lung [26], and breast [21, 27]. However, whether ALT inhibits OSCC progression and the underlying mechanism remains elusive. We aimed to evaluate the effects of ALT on OSCC and the involved mechanisms. The results revealed that ALT elicited OSCC cell death via ROS-mediated mitochondrial impairment and suppression of dynamin-related protein 1 (Drp1) phosphorylation. Our findings provide evidence that ALT is a promising therapeutic agent against OSCC. Furthermore, targeting mitochondria and Drp1 may be promising treatment modalities against OSCC.

## Methods

### Materials

The cell culture medium was from Life Technologies (Grand Island, NY, USA). Antibodies against Drp1 (sc-271583, Santa Cruz, CA, USA) and translocase of the outer membrane 20 (TOM20) (sc-17764, Santa Cruz, CA, USA), the phosphorylated-Drp1 (p-Drp1) antibody (AF8470, Affinity Biosciences, USA), the voltage-dependent anion channel 1 (VDAC1) antibody (ab14734, Abcam, Cambridge, UK), GAPDH (10494-1-AP, Proteintech Group, China) and secondary antibody (SA00001-2, Proteintech Group, China) were used. ALT (A114070, Aladdin Industrial Corporation, China) and *N*-acetyl-cysteine (NAC) (A7250, Sigma, USA) were applied.

### Cell culture and treatment

The human OSCC cells, including CAL27 and SCC9 cells, were from Wuhan University. CAL27 cells were cultured in DMEM and SCC9 cells were cultured in F-12K medium. Cells were cultured at 37 °C with 5% CO_2_. When the cells achieved 80% confluency, they were passaged and digested with trypsin. Cultured cells were treated with ALT of different concentrations and duration time. For experiments with NAC, the cells were pre-treated with 5 mM NAC for 2 h, according to the previous studies [28, 29].

Drp1 overexpressed plasmids were transfected into cells to explore the role of Drp1 in OSCC progression. The cells were inoculated into a 6-well plate and transfected with control and Drp1 overexpressed plasmids. All transfections were performed using Lipofectamine 3000 (L3000008, Invitrogen, USA) according to the manufacturer’s instructions [30]. Transfection efficiency was confirmed by western blot.

### Cell viability assay

We adopted the Methyl thiazolyl tetrazolium (MTT) assay to assess the cell viability. Cells were seeded in a 96-well plate at a density of 4500 cells/well in a volume of 100 μL/well. After incubating with MTT, dimethyl sulfoxide was added to the cells to dissolve the formazan crystals. The spectrophotometric absorbance was measured at 490 nm. The average absorbance of the control DMSO group was set at 100%, and the absorbance ratio reflected the cell viability.

### Western blot analysis

Cells were seeded in a 6-well plate at 2 × 10^5^ cells/well density and cultured under different conditions. Total proteins were collected using RIPA buffer (P0013, Beyotime, China), the concentrations of which were determined using the Bradford protein assay kit (23236, Thermo Fisher Scientific, USA). During the electrophoresis, we loaded 60–80 µg proteins in each slot in the SDS gel. Protein gels were blotted using the Trans-Blot Turbo transfer apparatus and PVDF Midi transfer packs (Bio-Rad). After blocking with 5% non-fat milk in TBST, blots were incubated with the primary antibodies with anti-p-Drp1 (1:1000), anti-Drp1 (1: 1000), and anti-GAPDH (1:20,000). After further incubation with secondary antibodies, blots were developed with ECL substrate (32209, Thermo Fisher Scientific, USA). NIH Image J software (https://imagej.nih.gov/ij/) detected the immunoreactive band relative to the optical density.

### Determination of intracellular ROS and RNS

2,7-Dichlorofluorescein diacetate (DCFH-DA) (S0033, Beyotime, China) was used to evaluate the intracellular ROS production. 3-amino,4-aminomethyl-2′,7′-fluorescein diacetate (DAF-FM DA) (S0019, Beyotime, China) was used to detect intracellular RNS level [31]. After treatment, the cells were stained with either DCFH-DA or DAF-FM DA for 20 min and rinsed with PBS. Images were taken by fluorescence microscope (Nikon, Japan) and evaluated by NIH Image J software (https://imagej.nih.gov/ij/).

### Mitochondria functional and morphology imaging assays

Cells were incubated with MitoSOX red to detect mitochondrial ROS (mtROS). Additionally, mitochondrial membrane potential (MMP) was evaluated with the tetramethylrhodamine methyl ester dye (TMRM). We detected the fluorescence signals of MitoSOX (M36006, Thrmofisher, USA), TMRM (I34361, Thrmofisher, USA), and MitoGreen (M36008, Thrmofisher, USA) at the excitation/emission wavelengths of 357/410 nm, 548/574 nm, and 491/513 nm. Fluorescence signals were measured using NIH Image J software.

### Colony outgrowth assay

Crystal violet (0.5% in 25% methanol) was used to stain the cells under different treatment conditions. A microscope (Olympus, Japan) was used to record the images. The colony formation areas were evaluated by Image J software (https://imagej.nih.gov/ij/).

### Annexin V-FITC/propidium iodide (PI) staining assay

An Annexin V-FITC/PI (C1062, Beyotime, China) apoptosis detection kit was adopted to detect the apoptotic rate. After trypsin digestion without ethylene diamine tetraacetic acid, the cells were collected by centrifugation and re-suspended. Annexin V-FITC and PI were added to the cell suspension in an ice bath. These staining conditions were examined by flow cytometer (CytoFLEX; Beckman Coulter, USA).

### Adenosine triphosphate (ATP) synthesis assays

ATP is primarily produced through oxidative phosphorylation in mitochondria and thus represents an essential indicator of mitochondrial function [32]. The CellTiter-Glo Assay evaluated ATP production. 100 µL of ATP detection buffer was added into each well. The luminescence was measured after 10 min of incubation, and the ATP level was normalized based on protein concentration.

### Immunohistochemistry staining

The specimens were formalin-fixed, paraffin-embedded, and cut into sheets of 4 µm. Gradient alcohol dehydrated the tissue slides after they were dewaxed with xylene. The sections were added with diluted primary antibody against p-Drp1 (1:200), Drp1 (1:200), TOM20 (1:200), and VDAC1 (1:200). Nuclei were counterstained with hematoxylin and then sealed in neutral rubber. In the final step, the sections were viewed under a light microscope.

### Patient samples and ethics

Wenzhou Medical University’s Research Ethics Committee approved protocol KY2022-R156 for this clinical-laboratory study. From October 2020 to December 2021, samples were obtained from 20 paired cancer tissues and adjacent tissues from patients at the Department of Oral and Maxillofacial Surgery, the First Affiliated Hospital of Wenzhou Medical University. Inclusion criteria were: (1) Patients complied with the Head and Neck Cancers Clinical Practice Guidelines [33]. (2) None of the patients had previously been treated with chemotherapy or radiotherapy. (3) The patients have no other malignancy or autoimmune diseases.

### Bioinformatics method

The RNA-seq transcriptome data of 500 head and neck squamous cell carcinoma tissues, 44 adjacent normal tissues and corresponding clinical data were obtained from the Cancer Genome Atlas (TCGA) (http://cancergenome.nih.gov/) database. MitoCarta2.0 documented the biological pathways and gene sets related to mitochondrial metabolism and thus was used in our study. Single-sample gene set enrichment analysis was used to calculate the infiltrating scores of mitochondrial-related metabolic pathways based on the above information. A protein–protein interaction (PPI) network was constructed using the Interacting Gene Retrieval Search tool (https://string-db.org/) to evaluate the interactions of mitochondrial metabolic pathways-related genes (MMPRGs). MMPRGs with |log Fold Change| > 0.5 and an adjusted *p-value* < 0.05 were identified as differentially expressed MMPRGs. Genes were further evaluated for prognostic significance with univariate cox regression analysis.

### Statistical analyses

Data analysis was performed with GraphPad Prism 8.0. Analyses of variance were performed to evaluate the data, expressing it as mean ± SD. Statistics were significant when *p* < 0.05. Six photographic fields were detected from all the figures and used for analysis. Each experiment was repeated three times. All bioinformatic analyses were conducted by packages and appropriate scripts in R (version 4.2.1) (http://www.R-project.org).

## Results

### ALT exhibited anti-proliferative and pro-apoptotic effects on OSCC cells

**Fig. 1:**
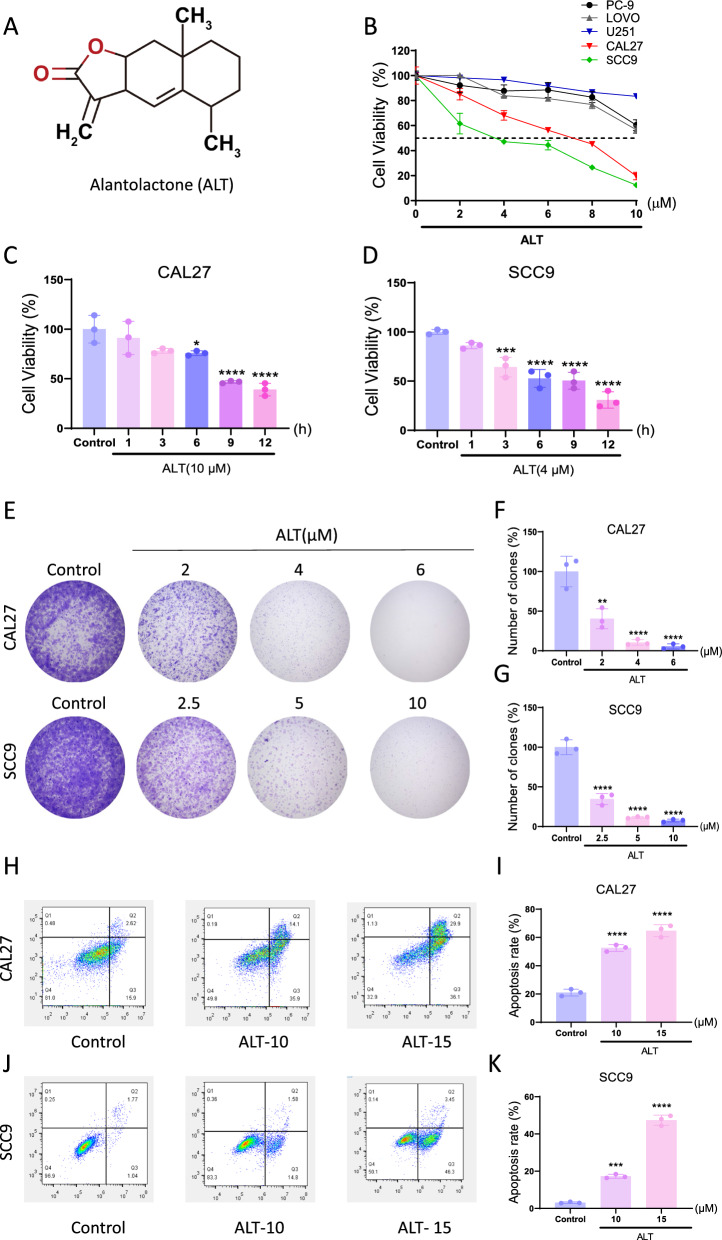
ALT inhibited cell viability and triggered OSCC cell apoptosis. **A** Chemical structure of ALT. **B** The inhibitory effects of ALT on PC-9, LOVO, U251, CAL27, and SCC9 cells. **C**, **D** Cell viability was measured in CAL27 and SCC9 cells after being treated with ALT for 24 h. **E**, **F** Cell viability was measured in CAL27 and SCC9 cells after being treated with ALT for indicated periods. **G**–**I** CAL27 and SCC9 cells were seeded into 6-well plates and then treated with various dosages of ALT as indicated. The number of colonies was assessed and quantified by crystal violet staining. **J**–**M** Flow cytometry analysis after Annexin V-FITC/PI double staining. (*p < 0.05, **p < 0.01, ***p < 0.001, ****p < 0.0001 versus the Control group)

Alantolactone (ALT) is a natural sesquiterpene lactone isolated from *Inula helenium* (Fig. 1A). We examined the effects of ALT on various types of cancer cells, including non-small cell lung cancer cell line PC-9, human LoVo colon cancer line, human glioblastoma cell lines U251, and the OSCC cell lines with CAL27 and SCC9 cells. The results showed that the OSCC cells were more sensitive to ALT than the other cancer subtypes (Fig. 1B), suggesting the strong inhibitory effect of ALT on OSCC. We further investigated the effects of ALT on CAL27 and SCC9 cell lines at different time intervals. ALT reduced the cell viability in a time-dependent way, with lower viability early at 12 h than the control group (Fig. 1C, D). In addition, ALT-treated cells formed smaller and fewer clones than their control counterparts in colony outgrowth assays (Fig. 1E–G). Taken together, ALT inhibited cell proliferation and colony formation abilities, which are essential for the formation of OSCC. We further explored the impact of ALT on the cell apoptosis rate of CAL27 and SCC9 cells. ALT increased the apoptotic rate of CAL27 and SCC9 cells, respectively, from 18.52 to 66% and from 2.81 to 49.75% (Fig. 1H–K).

### ROS regulation was essential in the preventive effects of ALT against OSCC cells

**Fig. 2:**
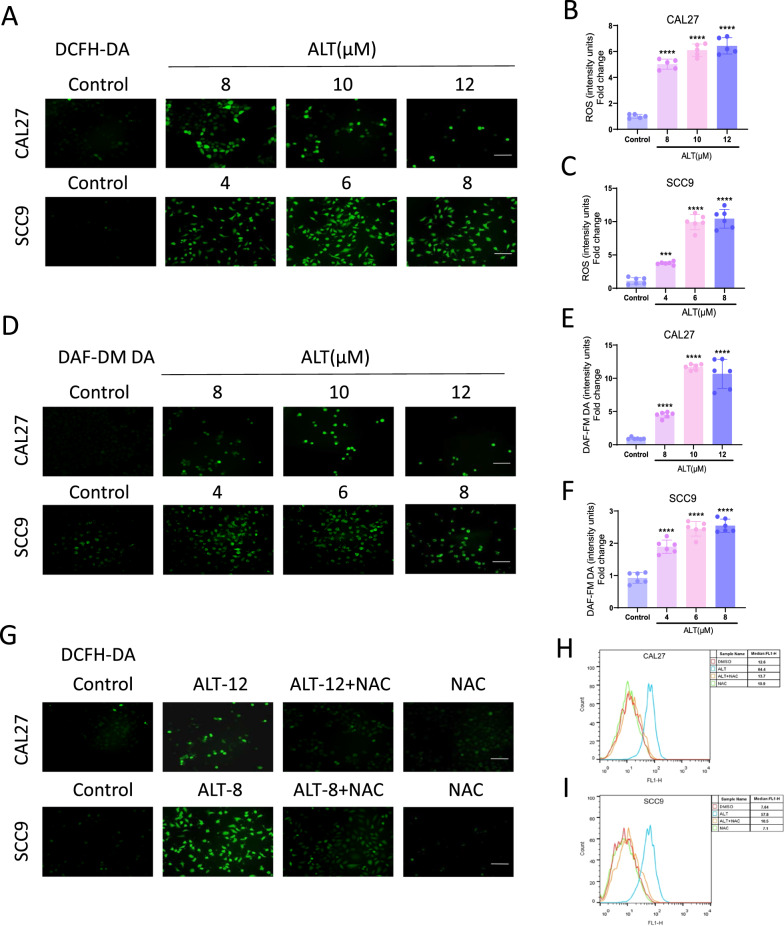
ROS regulation played a critical role in the preventive effect of ALT against OSCC cells. **A**–**C** Representative DCFH-DA fluorescent images in CAL27 and SCC9 cells treated with ALT. Mean optical density analysis of cellular DCFH-DA staining (green fluorescence; a ROS production indicator). Scale bar = 100 µm. **D**–**F** Representative DAF-FM DA fluorescent images in CAL27 and SCC9 cells treated with ALT. Mean optical density analysis of cellular DAF-FM DA staining (green fluorescence; an indicator of production of RNS). Scale bar = 100 µm. **G**–**I** Representative DCFH-DA fluorescent images in CAL27 and SCC9 cells treated with ALT with or without NAC. For experiments with NAC, the cells were pre-treated as with 5 mM NAC for 2 h. 12 µM ALT was used to treat CAL27 cells, and 8 µM ALT was added to SCC9 cells. Scale bar = 100 µm. (***p < 0.001, ****p < 0.0001 versus the Control group, ^####^*p* < 0.0001 versus the ALT group)

**Fig. 3:**
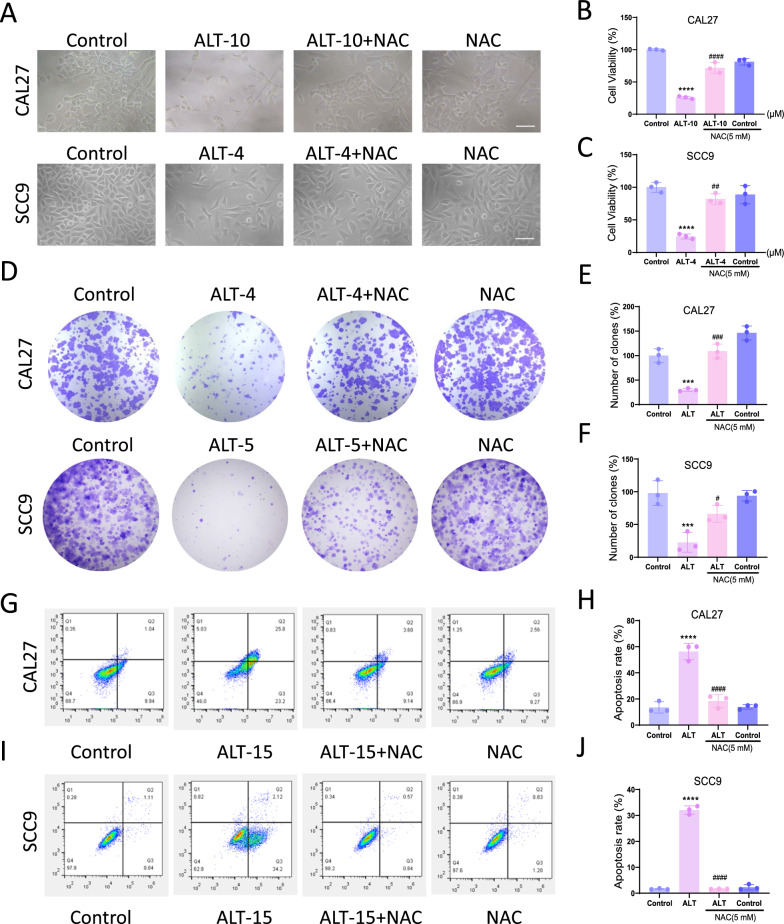
ROS regulation played a critical role in the preventive effect of ALT against OSCC cells. **A**–**C** Cell morphology observed by phase contrast microscope and cell viability analysis (scale bar = 100 µm). **D**–**F** The number of colonies assessed and quantified by crystal violet staining. **G**–**J** Flow cytometry-based Annexin V-FITC/PI labeling of apoptotic cells. We used 15 µM ALT to treat CAL27 cells and SCC9 cells. (**p < 0.01, ***p < 0.001, ****p < 0.0001 versus the Control group, ^#^*p* < 0.05, ^##^*p* < 0.01, ^###^*p* < 0.001, ^####^*p* < 0.0001 versus the ALT group)

To check whether ALT affects CAL27 and SCC9 cells via the regulation of OS, we measured the cellular total ROS level with the fluorescent probe DCFH-DA. ROS levels increased dose-dependently after ALT treatment (Fig. 2A–C). We further measured the RNS level as an additional readout of OS through DAF-FM DA. ALT dose-dependently increased the RNS levels in CAL27 and SCC9 cells (Fig. 2D–F). We further used NAC, a ROS scavenger, to identify whether ROS mediates the anticancer property of ALT. Compared with the cells only treated with ALT, a combination of NAC pretreatment and ALT treatment resulted in significantly lower ROS production in CAL27 and SCC9 cells, as confirmed both by DCFH-DA staining and flow cytometry (Fig. 2G–I). Quantitative analysis of MTT and colony outgrowth assay also showed that cells cotreated with NAC and ALT presented a higher proliferation rate than the lean ALT treatment group (Fig. 3A–F). Furthermore, NAC treatments reduced ALT-induced cell apoptosis (Fig. 3G–J). These results strongly substantiated that ROS regulation played crucial roles in the inhibitory effects of ALT on CAL27 and SCC9 cells.

### Mitochondrial abnormalities participated in the inhibitory effect of ALT against OSCC cells

**Fig. 4:**
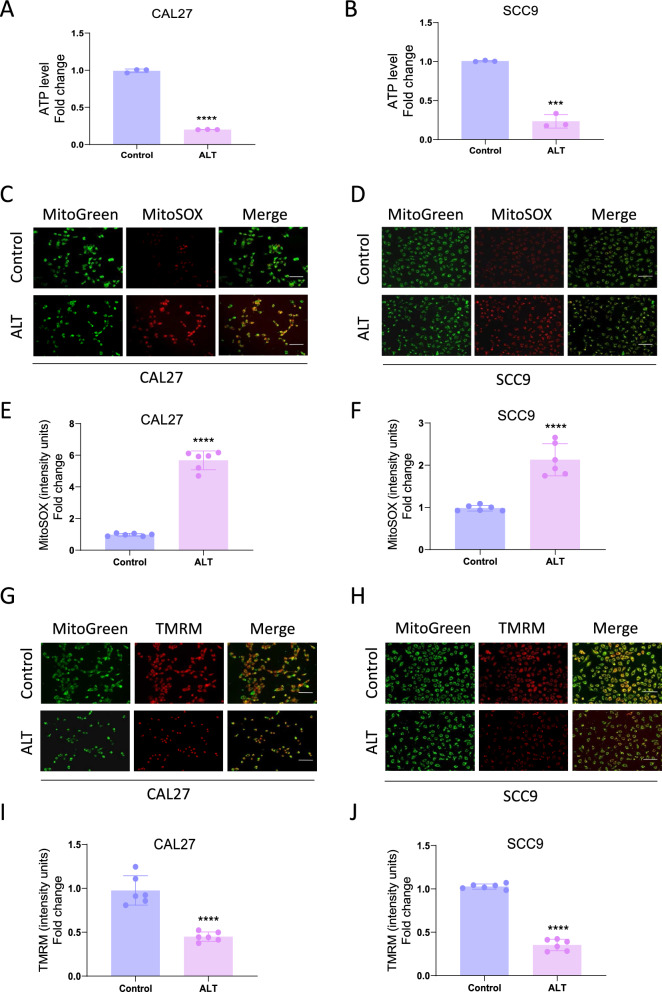
Mitochondrial abnormalities were involved in the inhibitory effect of ALT against OSCC cells. **A**, **B** ATP production in the indicated groups. 10 µM ALT was used to treat CAL27 cells, and 4 µM ALT was added to SCC9 cells. **C**–**F** Representative images showing MitoSOX staining and quantification in the indicated groups. 12 µM ALT was used to treat CAL27 cells, and 8 µM ALT was added to SCC9 cells. Scale bar = 100 µm. **G**–**J** Representative images showing TMRM staining and quantification in the indicated groups. 12 µM ALT was used to treat CAL27 cells, and 8 µM ALT was added to SCC9 cells. Scale bar = 100 µm. (**p < 0.01, ***p < 0.001, ****p < 0.0001 versus the Control group)

Regarding the close relationship between ROS and mitochondria, it is necessary to investigate whether mitochondria play a role in the inhibitory effect of ALT on OSCC. ALT treatment decreased the ATP levels in CAL27 and SCC9 cells (Fig. 4A, B). To further explore whether ALT-induced ROS production was derived from mitochondria, we used MitoSOX to indicate the mtROS production. The results showed that after ALT treatment, mtROS production in CAL27 and SCC9 cells was vitally increased (Fig. 4C–F). Besides, TMRM staining indicated that ALT markedly decreased MMP (Fig. 4G–J). These results suggested that ALT induced remarkable mitochondrial impairment, as indicated by increased mtROS level and MMP loss.

**Fig. 5:**
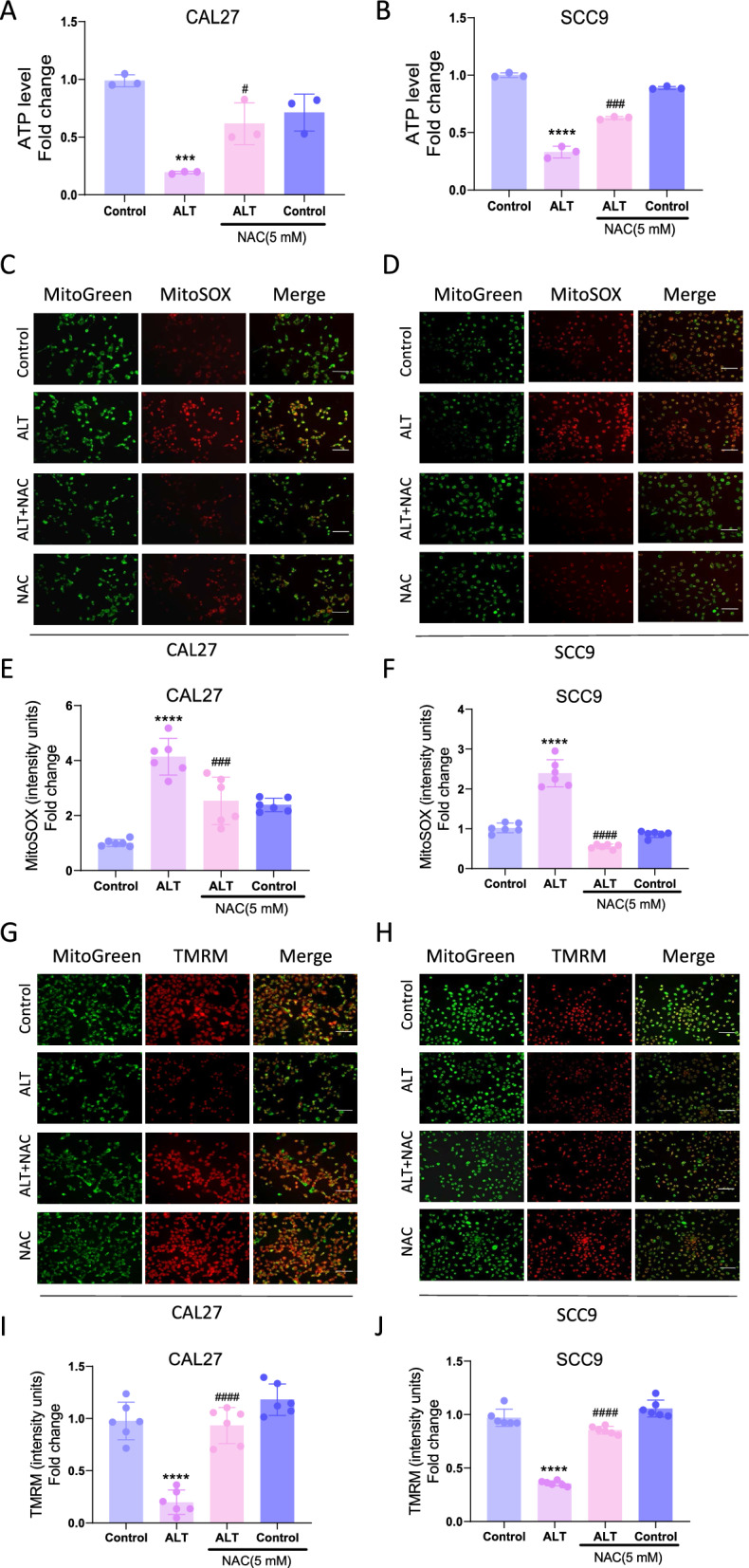
Mitochondrial abnormalities were involved in the inhibitory effect of ALT against OSCC cells. **A**, **B** ATP production in the indicated groups. 10 µM ALT was used to treat CAL27 cells, and 4 µM ALT was added to SCC9 cells. **C**–**F** Representative images showing MitoSOX staining and quantification in the indicated groups. 12 µM ALT was used to treat CAL27 cells, and 8 µM ALT was added to SCC9 cells. Scale bar = 100 µm. **G**–**J** Representative images showing TMRM staining and quantification in the indicated groups. 12 µM ALT was used to treat CAL27 cells, and 8 µM ALT was added to SCC9 cells. Scale bar = 100 µm. (***p < 0.001, ****p < 0.0001 versus the Control group, ^#^*p* < 0.05, ^###^*p* < 0.001, ^####^*p* < 0.0001 versus the ALT group)

We further applied NAC to probe the specific role of ROS in regulating ALT-elicited mitochondrial impairment. NAC dramatically reversed the ALT-elicited ATP depletion in CAL27 and SCC9 cells (Fig. 5A, B). Furthermore, ALT increased the mtROS production, which was reversed by NAC cotreatment. These results suggested that ALT-induced intracellular ROS production was mainly derived from mitochondria (Fig. 5C–F). Moreover, Cells treated with NAC and ALT had significantly higher MMP levels than those only treated with ALT, indicating that NAC reversed the MMP loss induced by ALT (Fig. 5G–J). These experiments revealed that ROS-related mitochondrial abnormalities played a crucial role in the ALT-elicited CAL27 and SCC9 cell apoptosis.

### Mitochondrial regulatory proteins were associated with OSCC progression and the inhibitory effect of ALT against OSCC cells

**Fig. 6:**
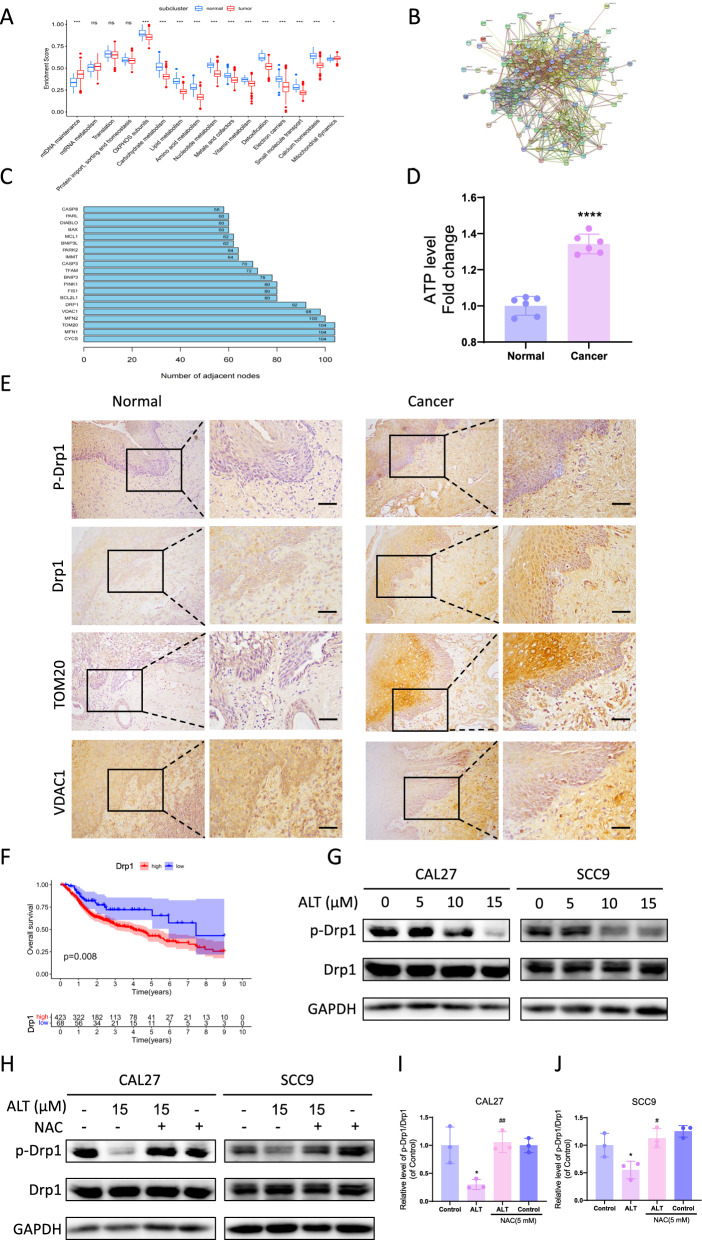
Mitochondrial regulatory proteins associated with OSCC progression and inhibitory effect of ALT against OSCC cells. **A** TCGA database evaluated expression patterns across diverse head and neck squamous carcinoma and normal tissues. **B** Cancer protein target interaction network. **C** Protein interaction relationship histogram. **D** ATP synthesis detected in the indicated groups. **E** Images of immunohistochemistry staining for mitochondrial regulating proteins in adjacent non-cancer tissues and OSCC tissues. Scale bar = 50 µm. **F** Prognostic graphs illustrating the impact of Drp1 on overall survival in TCGA. **G**, **H** Western blot band of Drp1 protein expression after different treatments. **I**, **J** p-Drp1 level expressed relative to Drp1 level. (****p < 0.0001 versus the Normal tissue group, *p < 0.05 versus the Control group, ^#^*p* < 0.05, ^##^*p* < 0.01 versus the ALT group)

To further explore the possible mitochondrial pathways involved in OSCC progression, we evaluated the gene expression patterns across diverse human OSCC and normal tissues with the TCGA database. Results showed that compared to the control group, mtDNA maintenance and mitochondrial dynamics were the two most differentially expressed pathways increased in the cancer tissues. Moreover, the pathways, such as amino acid metabolism and OXPHOS subunits, decreased significantly in the OSCC cancer tissues, which needs further exploration (Fig. 6A). Regarding the remarkable difference in mtDNA maintenance and mitochondrial dynamics between the normal and OSCC patients, we next focused on the possible mitochondrial regulating proteins involved in OSCC progression. Based on the STRING database, a PPI network of differentially expressed mitochondrial and mtDNA genes were constructed (Fig. 6B), and the top 20 proteins were obtained (Fig. 6C). Among the top 20 proteins, Drp1 (alias DNM1L), TOM20 (alias TOMM20), and VDAC1 differed significantly in cancer progression. Therefore, we further focused on the role of Drp1, TOM20, and VDAC1 in OSCC development. We collected cancer and normal tissues from 20 OSCC patients during surgery. ATP levels were strikingly higher in the cancer tissues than in the normal tissues (Fig. 6D). This result indicated that mitochondria played a crucial role in OSCC, as mitochondria are the primary source of ATP production. In OSCC cancer tissues, p-Drp1, Drp1, and TOM20 expressions increased significantly compared with normal tissues. Furthermore, the p-Drp1/Drp1 level increased significantly in the cancer samples (Additional file 1: Fig. S1). By contrast, the VDAC1 level was lower in OSCC cancer tissues than in normal tissues (Fig. 6E). Also, TCGA data revealed that OSCC patients with low Drp1 expression in cancer tissues had a higher overall survival rate than those with high Drp1 expression (p = 0.008) (Fig. 6F). More importantly, we explored whether Drp1 regulation participated in the inhibitory effects of ALT on OSCC cells. The results revealed that ALT dose-dependently downregulated the Drp1 phosphorylation level (Fig. 6G). We further investigated to explore the association between ROS generation and Drp1 dephosphorylation in the inhibitory effect of ALT on OSCC. The results confirmed that the ALT-induced decline of Drp1 dephosphorylation was reversed by NAC (Fig. 6H–J), indicating that ALT inhibited OSCC cell apoptosis by suppressing ROS-related Drp1 dephosphorylation.

### Drp1 participated in the inhibitory effect of ALT against OSCC cells

**Fig. 7:**
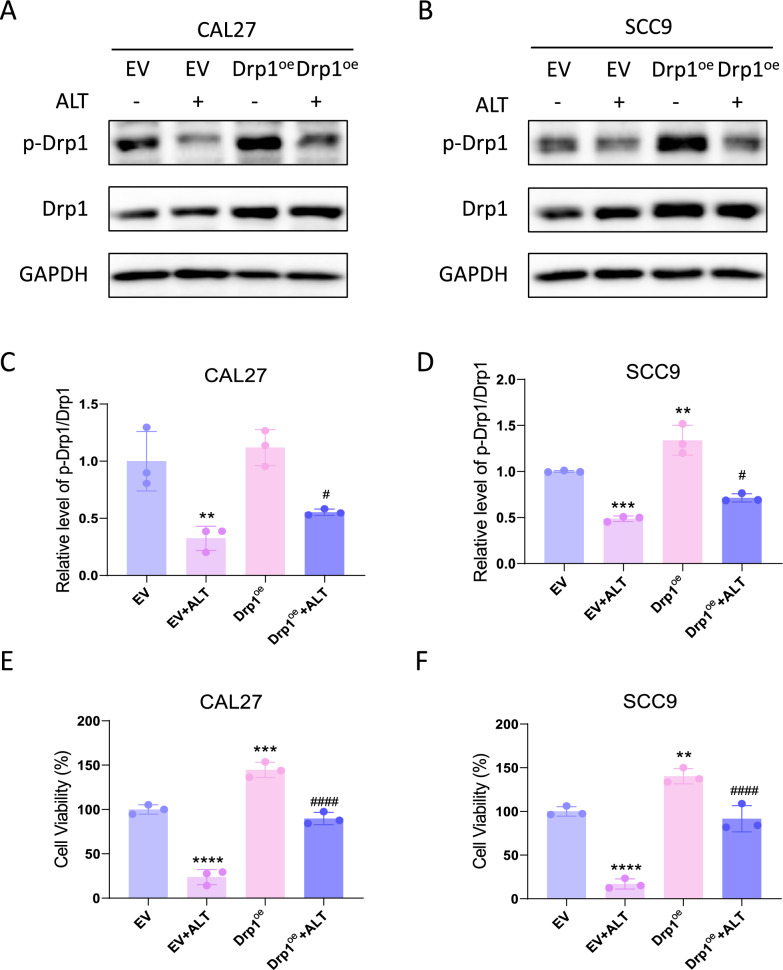
Drp1 is an important target of ALT against OSCC cells. **A**, **B** Western blot band of Drp1 protein expression after different treatments. We used 15 µM ALT to treat CAL27 cells and SCC9 cells. **C**, **D** p-Drp1 level relative to Drp1 level. **E**, **F** Cell viability was measured after being treated with ALT for 24 h. We used 10 µM ALT to treat CAL27 cells and 4 µM to treat SCC9 cells. (**p < 0.01, ***p < 0.001, ****p < 0.0001 versus the EV group, ^#^*p* < 0.05, ^####^*p* < 0.0001 versus the EV + ALT group)

**Fig. 8:**
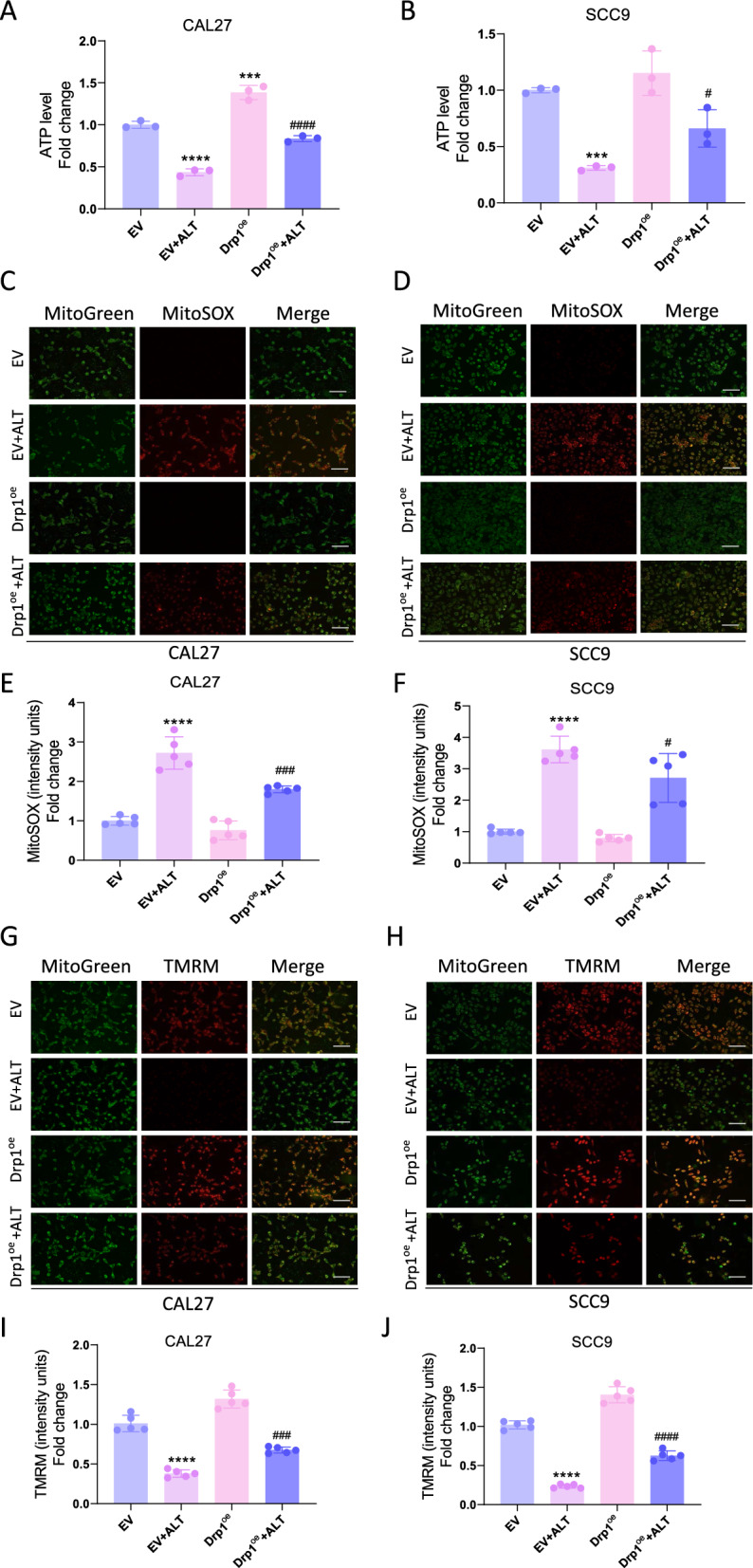
High expression of Drp1 reversed the abnormal mitochondrial effect of ALT on OSCC cells. **A**, **B** ATP production in the indicated groups. 10 µM ALT was used to treat CAL27 cells, and 4 µM ALT was added to SCC9 cells. **C**–**F** Representative images showing MitoSOX staining and quantification in the indicated groups. 12 µM ALT was used to treat CAL27 cells, and 8 µM ALT was added to SCC9 cells. Scale bar = 100 µm. **G**–**J** Representative images showing TMRM staining and quantification in the indicated groups. 12 µM ALT was used to treat CAL27 cells, and 8 µM ALT was added to SCC9 cells. Scale bar = 100 µm. (***p < 0.001, ****p < 0.0001 versus the EV group, ^#^*p* < 0.05, ^###^*p* < 0.001, ^####^*p* < 0.0001 versus the EV + ALT group)

Drp1 overexpression abolished the reduced Drp1 phosphorylation by ALT (Fig. 7A–D) and promoted the cell viability reduced by ALT (Fig. 7E, F). Drp1 overexpression also reversed the mitochondrial dysfunction induced by ALT, with increased ATP level (Fig. 8A, B), decreased ROS production (Fig. 8C–F), and increased mitochondrial membrane potential (Fig. 8G–J) compared to the control group.

## Discussion

In terms of malignancy rate and incidence, OSCC is one of the malignant oral carcinomas. Traditional therapies against OSCC, including surgery, chemotherapy, and radiotherapy, are sometimes ineffective because of drug resistance and side effects [7]. Hence, identifying novel and effective treatments is imperative. Mitochondria-targeting therapies have been proven effective in treating various cancers including the colon [14] and breast [15] cancers. However, studies on mitochondrial-targeting therapy for OSCC are limited and need further exploration. Our findings confirmed that ALT potently restrained the proliferation and triggered apoptosis of OSCC cells by inducing mtROS production, reducing MMP, and regulating Drp1 dephosphorylation.

OS is characterized by redox imbalances caused by the overproduction of ROS [9], which plays a vital role in developing various diseases [34]. In physiological conditions, the ROS accumulated in cells is rapidly eliminated by antioxidant enzymes. However, ROS are overproduced in pathological conditions, causing oxidative damage to biomolecules and worsening the cellular oxidative status [35]. Excessive ROS contribute to cancer development and can be used as therapeutic targets [36]. In our study, ALT reduced cell viability and enhanced apoptotic rate of OSCC cells by promoting ROS generation. Thus, ROS may be a promising therapeutic target for OSCC.

Mitochondria play a crucial part in cancer formation and progression [17]. Mitochondria have been recognized as the major source of ROS, which plays a critical role in cancer progression. Numerous studies have clarified the differences in mitochondria between normal and cancer cells, which might be used for cancer treatment [13–15, 21, 37]. Mitochondria-targeted therapies have been proven to be effective in preventing various cancers [38]. However, their application in OSCC treatment needs further exploration. In this study, the bioinformatics analysis revealed that mtDNA maintenance and mitochondrial dynamics were closely associated with OSCC. The expressions of mitochondrial proteins, including p-Drp1, Drp1, and TOM20, were notably higher in the cancer tissues of OSCC patients compared with the normal tissues. However, the expression of VDAC1 in OSCC tissues was lower than that in normal tissues. The specific role of these mitochondrial proteins in OSCC needs further exploration. Additionally, we elucidated that NAC effectively suppressed the mitochondrial membrane depolarization and mtROS production induced by NAC. NAC also blocked the ALT-elicited ROS production and cytotoxicity in CAL27 and SCC9 cells. These findings further indicated that ALT promoted CAL27 and SCC9 cell apoptosis by promoting ROS production and mitochondrial impairment.

ALT contains a wide variety of pharmacological properties, including anticancer [20], antibacterial [18], and anti-inflammatory activities [18]. Additionally, studies have proved that ALT prevented cancer progression via glutathione depletion, ROS induction [39], and mitochondrial impairment [21]. Our results also revealed that ALT induced cell death through the enhancement of ROS-mediated mitochondrial dysfunction in OSCC, implying mitochondrial-targeting therapy as a promising therapy strategy against OSCC. Drp1, the primary regulator of mitochondrial function and dynamics [40], is essential for developing various cancers [41]. The results illustrated that the p-Drp1 level increased vitally in the cancer tissues compared to the adjacent normal tissues. According to the bioinformatics analysis, OSCC patients with low Drp1 expressions had better overall survival than those with high Drp1 levels. Immunohistochemical results further confirmed that p-Drp1 and Drp1 increased significantly in OSCC cancer tissues compared to normal tissues. Furthermore, the cancer samples presented enhanced p-Drp1/Drp1 level, suggesting the essential role of Drp1 level and Drp1 phosphorylation in OSCC progression. In addition, ALT inhibited the Drp1 phosphorylation in OSCC cells. Our results further revealed that Drp1 overexpression abolished the reduced Drp1 phosphorylation by ALT and promoted the cell viability of ALT-treated cells. Drp1 overexpression also reversed the mitochondrial dysfunction induced by ALT. Taken together, ALT potentially induced OSCC cell apoptosis by downregulating Drp1 phosphorylation. ALT is a selective inhibitor of signal transducers and activators of transcription 3 (STAT3) [42]. Studies have shown that STAT3 is vital for Drp1 activation and mitochondrial fission [43–45]. Further studies are warranted to investigate whether STAT3 is an intermediate factor in the mitochondrial pathway mediated by ALT and Drp1.

To date, ALT has been used for treating various diseases through oral administration [21, 23–25]. However, the oral bioavailability of ALT is considerably low [46]. Nanostructured carriers entrapped with ALT have been used to improve bioavailability in treating cancers [47]. In most cases, OSCC mainly occurs at superficial sites [3]. Therefore, local applications of ALT-entrapped nanostructured gels or sprays represent promising treatment strategies for OSCC [48, 49].

Despite the critical results, this study has some limitations. Firstly, experiments were conducted only in CAL27 and SCC9 cells, while the normal cells were not involved. Further studies with normal cells are warranted. Additionally, the sample size was relatively small. More subjects should be enrolled to improve the reliability of our study. Finally, in vivo studies should be conducted to substantiate the in vitro results in future studies.

**Fig. 9:**
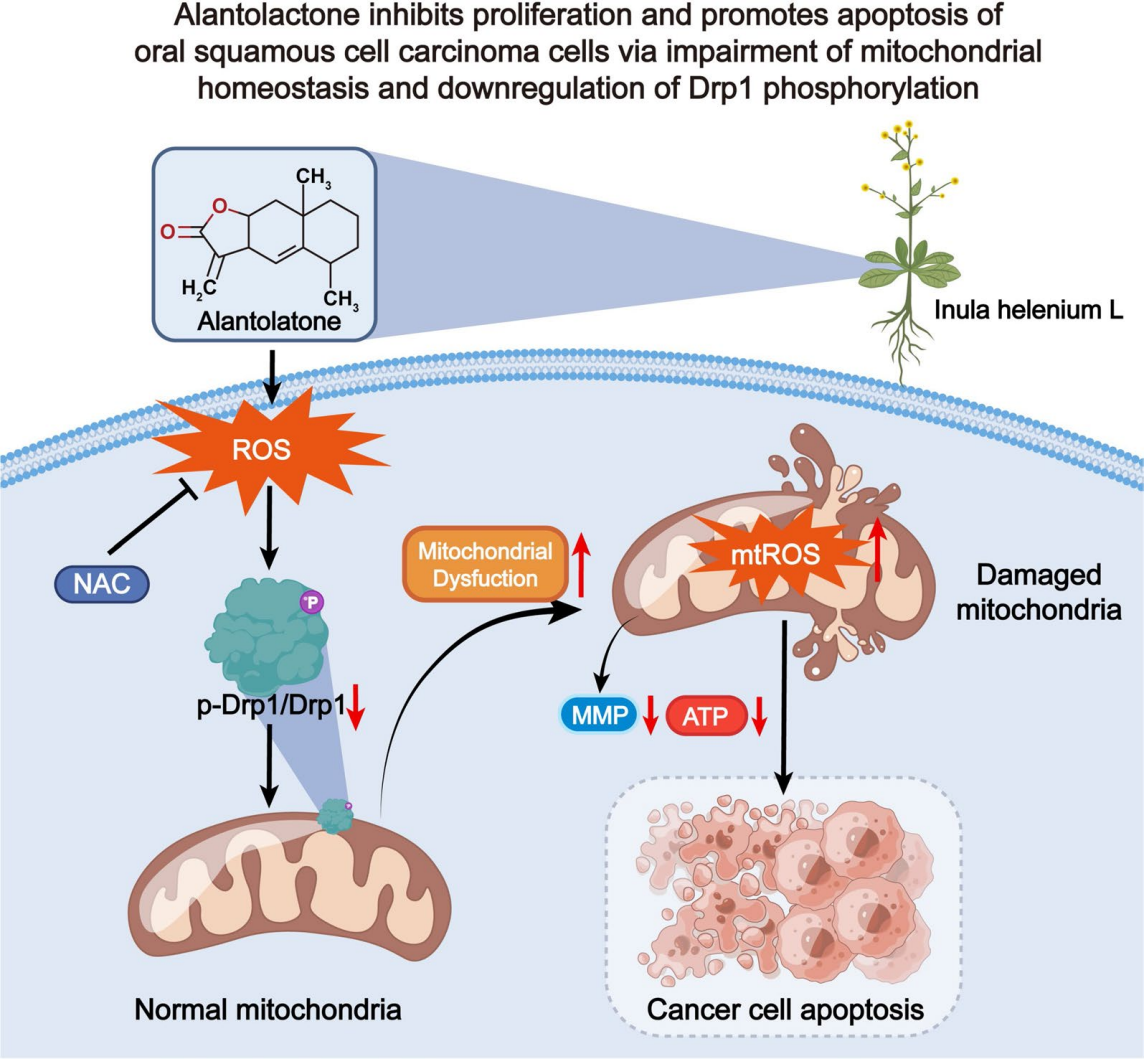
Diagram of molecular mechanism. Alantolactone inhibits proliferation and promotes apoptosis of oral squamous cell carcinoma cells via impairment of mitochondrial homeostasis and downregulation of Drp1 phosphorylation

In summary, ALT induced OSCC cell apoptosis via enhancement of ROS-dependent mitochondria impairment and down-regulation of Drp1 phosphorylation (Fig. 9). The results provide a solid basis for the application of ALT in treating OSCC, with Drp1 being a promising therapeutic target for OSCC.

## Conclusions

ALT induced OSCC cell injury via promoting mitochondrial dysfunction and downregulation of Drp1 phosphorylation. Taken together, these findings provide a novel paradigm for the ALT treatment against OSCC, with Drp1 being a promising therapeutic target for OSCC.

## Supplementary Information

**Additional file 1: Figure S1.** Statistical analysis of the level of mitochondrial proteins in OSCC cancer tissues.The corresponding statistical analysis of P-Drp1/GAPDH.The statistical analysis of Drp1/GAPDH.The statistical analysis of P-Drp1/Drp1.The statistical analysis of TOM20/GAPDH.The statistical analysis of VDAC1/GAPDH.

## Data Availability

The original contributions presented in the study are included in the article. Further inquiries can be directed to the corresponding authors.

## Abbreviations

ALT: Alantolactone
ATP: Adenosine triphosphate
DAF-FM DA: 4-Amino-5-methylamino-2′,7′-difluorofluorescein diacetate
DCFH-DA: 2,7-Dichlorodihydrofluorescein diacetate
Drp1: Dynamin-related protein 1
MMP: Mitochondrial membrane potential
MMPRGs: Mitochondrial metabolic pathways related genes
mtDNA: Mitochondrial DNA
mtROS: Mitochondrial reactive oxygen species
MTT: Methyl thiazolyl tetrazolium
NAC: *N*-Acetyl-cysteine
OS: Oxidative stress
OSCC: Oral squamous cell carcinoma
p-Drp1: Phosphorylated-Drp1
RNS: Reactive nitrogen species
ROS: Reactive oxygen species
STAT3: Signal transducers and activators of transcription 3
TCGA: The Cancer Genome Atlas
TMRM: Tetramethylrhodamine methyl ester
TOM20: Translocase of the outer membrane 20
VDAC1: Voltage-dependent anion channel 1
