# Supplementary figures and images for: Mitochondrial impairment and downregulation of Drp1 phosphorylation underlie the antiproliferative and proapoptotic effects of alantolactone on oral squamous cell carcinoma cells
Source: J Transl Med. 2023 May 18;21:328. doi: 10.1186/s12967-023-04188-2 (PMC10193726; doi:10.1186/s12967-023-04188-2)

A

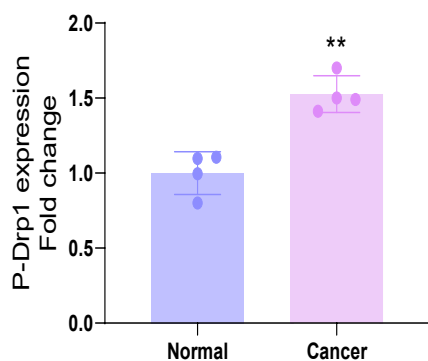

B

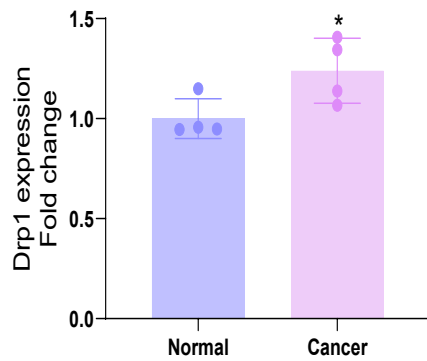

C

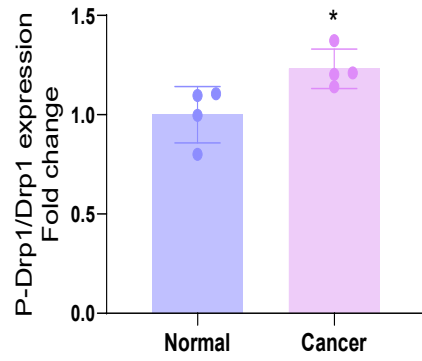

D

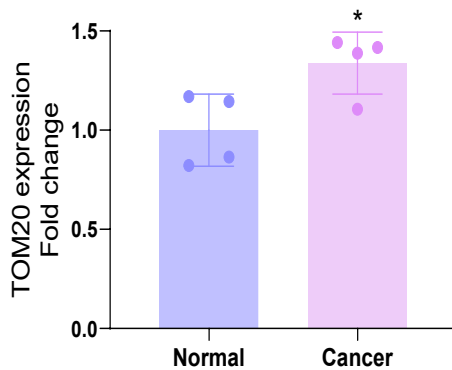

E

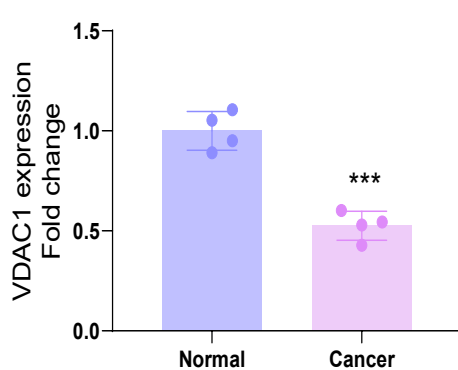

Supplement: Supplementary file 1 — Additional file 1: Figure S1. Statistical analysis of the level of mitochondrial proteins in OSCC cancer tissues.The corresponding statistical analysis of P-Drp1/GAPDH.The statistical analysis of Drp1/GAPDH.The statistical analysis of P-Drp1/Drp1.The statistical analysis of TOM20/GAPDH.The statistical analysis of VDAC1/GAPDH. [file 12967_2023_4188_MOESM1_ESM.pdf]
